# Gender imbalance in medical imaging datasets produces biased classifiers for computer-aided diagnosis

**DOI:** 10.1073/pnas.1919012117

**Published:** 2020-05-26

**Authors:** Agostina J. Larrazabal, Nicolás Nieto, Victoria Peterson, Diego H. Milone, Enzo Ferrante

**Affiliations:** ^a^Research Institute for Signals, Systems and Computational Intelligence sinc(i), Universidad Nacional del Litoral–Consejo Nacional de Investigaciones Científicas y Técnicas CONICET, Santa Fe CP3000, Argentina;; ^b^Instituto de Matemática Aplicada del Litoral, Universidad Nacional del Litoral–Consejo Nacional de Investigaciones Científicas y Técnicas, Santa Fe CP3000, Argentina;; ^c^Facultad de Ingeniería, Universidad Nacional de Entre Ríıos, Oro Verde CP3100, Argentina

**Keywords:** gendered innovations, deep learning, computer-aided diagnosis, medical image analysis, gender bias

## Abstract

Artificial intelligence (AI) systems for computer-aided diagnosis and image-based screening are being adopted worldwide by medical institutions. In such a context, generating fair and unbiased classifiers becomes of paramount importance. The research community of medical image computing is making great efforts in developing more accurate algorithms to assist medical doctors in the difficult task of disease diagnosis. However, little attention is paid to the way databases are collected and how this may influence the performance of AI systems. Our study sheds light on the importance of gender balance in medical imaging datasets used to train AI systems for computer-assisted diagnosis. We provide empirical evidence supported by a large-scale study, based on three deep neural network architectures and two well-known publicly available X-ray image datasets used to diagnose various thoracic diseases under different gender imbalance conditions. We found a consistent decrease in performance for underrepresented genders when a minimum balance is not fulfilled. This raises the alarm for national agencies in charge of regulating and approving computer-assisted diagnosis systems, which should include explicit gender balance and diversity recommendations. We also establish an open problem for the academic medical image computing community which needs to be addressed by novel algorithms endowed with robustness to gender imbalance.

Artificial intelligence (AI) influences almost every aspect of our daily life. The media articles we read, the movies we watch, even the driving road map we take are somehow influenced by these systems. In particular, the rise of AI in healthcare during the last few years is changing the way medical doctors diagnose, especially when dealing with medical images. AI systems cannot only augment the information provided by such images with useful annotations (1, 2), but they are also starting to take autonomous decisions by performing computer-aided diagnosis (CAD) (3, 4).

Although the interest in performing fair and unbiased evaluations of AI medical systems has existed since the 1980s (5), the ethical aspects of AI have gained relevance in the last few years. It has been shown that human bias, such as gender and racial bias, may not only be inherited but also amplified by AI systems in multiple contexts (6–9). For example, face recognition systems have been shown to exhibit accuracy disparities depending on gender and ethnicity, with darker-skinned females being the most misclassified group (10). This tendency of AI systems to learn biased models, which reproduce social stereotypes and underperform in minority groups, is especially dangerous in the context of healthcare (11, 12).

In recent years, the research community of gendered innovations has largely contributed to create awareness and integrate sex and gender analyses into all phases of basic and applied research (13). However, such assessment in the context of medical imaging and CAD remains largely unexplored. In this work, we perform a large-scale study that quantifies the influence of gender imbalance in medical imaging datasets used to train AI-based CAD systems. It is worth mentioning that most of the existing work dealing with imbalanced data in the context of deep learning focuses on cases where it is related to the target classes (14, 15). In our study, this would translate to an imbalance in terms of number of patients per pathology. However, note that, in this case, the imbalance is given by a demographic variable different from the target class: gender, which is generally neglected. Our results show that using gender-imbalanced datasets to train deep learning-based CAD systems may affect the performance in pathology classification for minority groups.

## Results and Discussion

**Fig. 1.**
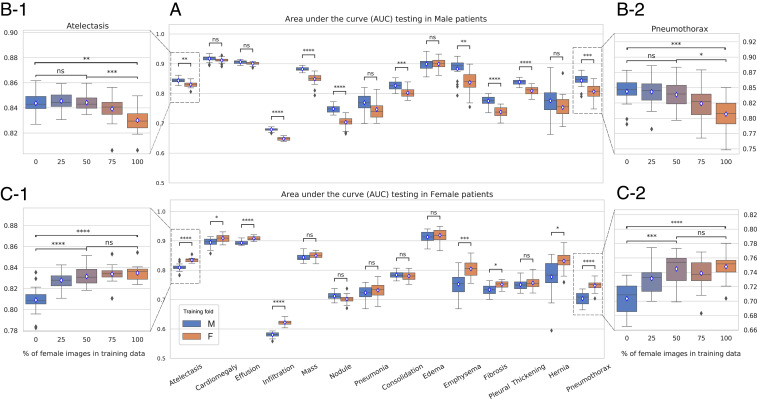
Experimental results for a DenseNet-121 (18) classifier trained with images from the NIH dataset (16, 19) for 14 thoracic diseases under different gender imbalance ratios. (*A*) The box plots aggregate the results for 20 folds, training with male-only (blue) and female-only (orange) patients. Both models are evaluated given male (*Top*) and female (*Bottom*) test folds. A consistent decrease in performance is observed when using male patients for training and female for testing (and vice versa). (*B* and *C*) AUC achieved for two exemplar diseases under a gradient of gender imbalance ratios, from 0% of female images in training data to 100%, with increments of 25%. In *B*, *1* and *2* show the results when testing on male patients, while, in *C*, *1* and *2* present the results when testing on female patients. Statistical significance according to Mann–Whitney *U* test is denoted by **** (P≤ 0.00001), *** (0.00001 <P≤ 0.0001), ** (0.0001 <P≤ 0.001), * (0.001 <P≤^,^ 0.01), and not significant (ns) (P>0.01).

A model based on deep neural networks, which achieves state-of-the-art results when diagnosing 14 common thoracic diseases using X-ray images (16), was implemented to perform CAD. We employed the area under the receiver operating characteristic curve (AUC) (17) to quantify its performance. Fig. 1 shows the experimental results obtained when training the classifier under different gender imbalance ratios. In Fig. 1*A*, the box plots aggregate the results for 20 experiments using fully imbalanced datasets. The blue boxes represent the performance for models trained only with male images, while orange boxes indicate training with female-only images. Both models are evaluated over male-only (Fig. 1 *A*, *Top*) and female-only (Fig. 1 *A*, *Bottom*) test images. A consistent decrease in performance is observed when using male patients for training and female for testing (and vice-versa). The same tendency was confirmed when evaluating three different deep learning architectures in two X-ray datasets with different pathologies.

We also explored intermediate imbalance scenarios, where both female and male patients were present in the training dataset but considering different proportions (0%/100%, 25%/75%, and 50%/50%). Fig. 1 *B* and *C* shows the average classification performance for two exemplar diseases, Pneumothorax and Atelectasis, under such gradient of gender imbalance ratios (indicated with the percentage of female patients used for training). We found that, even with a 25%/75% imbalance ratio, the average performance across all diseases in the minority class is significantly lower than a model trained with a perfectly balanced dataset. Moreover, we did not find significant differences in performance between models trained with a gender-balanced dataset (50% male and 50% female) and an extremely imbalanced dataset from the same gender. In other words, a CAD system trained with a diverse (and balanced) dataset achieved the best performance for both genders. Altogether, our results indicate that diversity provides additional information and increases the generalization capability of AI systems. Thereafter, it also suggests that diversity should be prioritized when designing databases used to train machine learning-based CAD systems.

Our study shows that gender imbalance in medical imaging datasets produces biased classifiers for computer-aided diagnosis based on convolutional neural networks (CNNs), with significantly lower performance in underrepresented groups. We provide experimental evidence in the context of X-ray image classification for such potential bias, aiming to raise the alarm not only within the medical image computing community but also for national agencies in charge of regulating and approving medical systems. As an example, let us take the US Food and Drug Administration. Even though they have released several documents related to the importance of gender/sex issues in the design and evaluation of clinical trials and medical devices (21), when looking at the specific guidelines to obtain the certification to market medical computer-aided systems (22, 23), there is no explicit mention of gender/sex as one of the relevant demographic variables that should describe the sampled population. Similar issues are observed in the medical imaging community. Albeit a few datasets provide this information at the subject level, most public datasets of similar characteristics do not contain gender/sex information at the patient level to date [e.g., the recent MIMIC-CXR (24) x-ray dataset or the Retinal Fundus Glaucoma Challenge (REFUGE) database of ophtalmological images (25), just to name a few]. The same tendency is observed in many of the datasets included in a recent analysis of 150 databases from grand challenges on biomedical image analysis (26), which provides recommendations for database and challenge design, where there is no explicit mention of the importance of sex/gender demographic information.

In general, it is well known that CNNs tend to learn representations useful to solve the task they are being trained for. When we go from male to female images (or vice versa), structural changes in the images appear, leading to a change in data distribution which explains the decrease in performance. Algorithmic solutions to such “domain adaptation” problems (27) should be engineered, especially in cases when it is difficult to obtain gender-balanced datasets [e.g., Autism Brain Imaging Data Exchange (ABIDE) I (28)].

## Materials and Methods

### Datasets.

We use the NIH Chest-XRay14 dataset (16, 19), which includes 112,120 chest X-ray images from 30,805 patients, labeled with 14 common thorax diseases (including hernia, pneumonia, fibrosis, emphysema, edema, cardiomegaly, pleural thickening, consolidation, mass, pneumothorax, nodule, atelectasis, effusion, and infiltration). Labeling was performed according to an automatic natural language processing analysis of the radiology reports. The dataset provides demographic information including the patient’s gender: 63,340 (56.5%) images for male and 48,780 (43.5%) images for female patients. Following the demographic variables reported in the original dataset publication (19), we used the term “gender” to characterize our imbalance study. However, given that some anatomical attributes are reflected in X-ray images, the term *sex* could be more accurate, according to the Sex and Gender Equity in Research guidelines (29). The CheXpert database (30) was also used to confirm that our observations generalize for different datasets. It contains 224,316 chest radiographs of 65,240 patients with diagnostic information (∼60% male and ∼40% female). The uncertainty labels included in CheXpert were interpreted as negative following the U-Zeros approach discussed in the original paper (30).

### Deep Learning Model.

Deep neural networks are machine learning methods with multiple abstraction levels, which compose simple but nonlinear modules transforming representations at one level into a representation at a higher, slightly more abstract level (31). A special type of deep neural network, known as CNNs, was used to implement the CAD system (19, 20). Results shown in Fig. 1 correspond to a Densely Connected CNN (DenseNet) architecture with 14 outputs, one for each disease (18).

We adopted a Keras implementation of the DenseNet-121 which has been shown to achieve state-of-the-art results in X-ray image classification (16). The network has 121 convolutional layers and a final fully connected layer producing a 14-dimensional output, after which we apply an element-wise sigmoid nonlinearity. A model pretrained on ImageNet (32) was used to initialize the network weights. We trained it end to end using Adam optimizer with standard parameters (β1 = 0.9 and β2 = 0.999), a batch size of 32, and an initial learning rate of 0.001 that was decayed by a factor of 10 each time the validation loss plateaued after an epoch. Additionally, we evaluated two other CNN architectures, the ResNet (33) and the Inception-v3 (34), confirming that our observations generalize for different neural models.

### Methodology.

Since images can be labeled with multiple diseases, we implemented an automatic method to construct random splits, which guarantees that male and female folds will have the same number of images per pathology. Given a frontal X-ray image, the CAD system predicts the presence or absence of the 14 thoracic diseases. Two models were trained in each experiment, one considering male-only datasets, while the other considered female-only training datasets. Intermediate imbalance scenarios were also analyzed, in which female and male images were presented in the training dataset at different proportions (0%/100%, 25%/75% and 50%/50%). To avoid other sources of bias, care was taken to guarantee, by training data construction, that male and female folds include the same number of pathological cases per class. For the NIH Chest-XRay14, every split included 48,568 images. For the CheXpert dataset, every split included 27,147 images. The same experiment was performed 20 times, using different random splits. In the testing phase, both models were evaluated in male and female patients separately. The classification performance was measured by the well-known AUC (17).

### Data Availability.

The NIH Chest-XRay14 dataset is publicly available at https://nihcc.app.box.com/v/ChestXray-NIHCC. The CheXpert dataset is publicly available at https://stanfordmlgroup.github.io/competitions/chexpert/. The source code of the original CNNs is publicly available at https://github.com/brucechou1983/CheXNet-Keras. The modified version of this code with our auxiliary scripts, the data splits used in our experiments, and the additional results for all of the CNN architectures in both datasets can be accessed at https://github.com/N-Nieto/GenderBias_CheXNet.
